# Ledipasvir/sofosbuvir for treatment-naive and treatment-experienced Chinese patients with genotype 1 HCV: an open-label, phase 3b study

**DOI:** 10.1007/s12072-018-9856-z

**Published:** 2018-04-10

**Authors:** Lai Wei, Qing Xie, Jin Lin Hou, Hong Tang, Qin Ning, Jun Cheng, Yuemin Nan, Lunli Zhang, Jun Li, Jianning Jiang, Brian McNabb, Fangqiu Zhang, Gregory Camus, Hongmei Mo, Anu Osinusi, Diana M. Brainard, Guozhong Gong, Zhuangbo Mou, Shanming Wu, Guiqiang Wang, Peng Hu, Yanhang Gao, Jidong Jia, Zhongping Duan

**Affiliations:** 1 Beijing Key Lab for Hepatitis C and Immunologic Liver Disease, Peking University Hepatology Institute, Peking University People’s Hospital, 11 Xizhimen S St, Xicheng District, Beijing, 100044 China; 2 Ruijin Hospital, Shanghai Jiaotong University, Shanghai, China; 3 Nanfang Hospital of Southern Medical University, Guangzhou, China; 4 West China Hospital, Sichuan University, Chengdu, China; 5 Tongji Hospital of Tongji Medical College, Huanzhong University of Science and Technology, Wuhan, China; 6 Beijing Ditan Hospital Affiliated to Capital Medical University, Beijing, China; 7 The Third Hospital of Hebei Medical University, Hebei, China; 8 The First Affiliated Hospital of Nanchang University, Nanchang, China; 9 The First Affiliated Hospital with Nanjing Medical University, Nanjing, China; 10 The First Affiliated Hospital of Guangxi Medical University, Guangxi, China; 11 Gilead Sciences, Inc., Foster City, USA; 12 The Second Xiangya Hospital of Central South University, Changsha, China; 13 Jinan Infectious Disease Hospital, Jinan, China; 14 Clinical Center of Shanghai Public Health, Shanghai, China; 15 Peking University First Hospital, Beijing, China; 16 The Second Affiliated Hospital of Chongqing Medical University, Chongqing, China; 17 The First Hospital of Jilin University, Changchun, China; 18 Beijing Friendship Hospital, Capital Medical University, Beijing, China; 19 Beijing You-An Hospital, Capital Medical University, Beijing, China

**Keywords:** China, Genotype 1, Hepatitis C virus, Ledipasvir, Sofosbuvir, Single-tablet regimen

## Abstract

**Background:**

Chronic hepatitis C virus (HCV) infection is a significant medical burden in China, affecting more than 10 million persons. In clinical trials and real-world settings, treatment with ledipasvir/sofosbuvir in patients with genotype 1 HCV infection resulted in high sustained virologic response rates. Ledipasvir/sofosbuvir may provide a highly effective, short-duration, single-tablet regimen for Chinese patients with HCV infection.

**Methods:**

Chinese patients with genotype 1 HCV infection who were HCV treatment naive or treatment experienced, without cirrhosis or with compensated cirrhosis, were treated with open-label ledipasvir/sofosbuvir for 12 weeks. The primary efficacy endpoint was sustained virologic response 12 weeks after completing treatment (SVR12). For treatment-naive patients, SVR12 was compared to a historical rate of 57%. The primary safety endpoint was adverse events leading to permanent discontinuation of study drug; serious adverse events were also evaluated. The presence of resistance-associated substitutions (RASs) was evaluated by viral sequencing.

**Results:**

All 206 enrolled patients achieved SVR12 (100%; 95% CI 98–100%), including 106 treatment-naive patients (100%; 95% CI 97–100%), which was superior to a historical SVR rate of 57% (*p* < 0.001). All patients with baseline NS5A and NS5B RASs (14 and 1% of patients, respectively) achieved SVR12. The most common adverse events were viral upper respiratory tract infection (17%), upper respiratory tract infection (14%), and cough (6%). There were no discontinuations due to adverse events; and no treatment-related serious adverse events were reported.

**Conclusion:**

Ledipasvir/sofosbuvir is a well tolerated and highly effective treatment for Chinese patients with genotype 1 HCV, regardless of prior treatment experience, cirrhosis status, or the presence of pretreatment RASs.

**Electronic supplementary material:**

The online version of this article (10.1007/s12072-018-9856-z) contains supplementary material, which is available to authorized users.

## Introduction

The prevalence of chronic hepatitis C virus (HCV) infection in China is estimated at 0.7%, with genotype 1b HCV accounting for approximately 57% of infections [1]. Until recently, the only treatment option for HCV-infected patients in China was pegylated interferon in combination with ribavirin. Interferon-based treatment regimens are poorly tolerated, and a large number of patients have remained untreated due to absolute and relative contraindications [2–4].

The introduction of direct-acting antiviral (DAA) therapy in China in 2017 has improved treatment options and outcomes for patients with HCV infection [5]. Three all-oral DAA regimens are approved for use in China: sofosbuvir, a potent selective inhibitor of HCV NS5B polymerase, in combination with ribavirin; and daclatasvir, NS5A replication complex inhibitor, in combination with either sofosbuvir or asunaprevir, NS3 protease inhibitor [6–8]. While these regimens have demonstrated improved sustained virologic response rates and safety profiles relative to interferon-based regimens [8, 9], they require either the use of ribavirin, with its associated hematologic, constitutional, and neuropsychiatric side effects [10] and additional concerns of genotoxicity and teratogenicity in younger patients; viral genotype subtyping and resistance profiling [4]; or extension of therapy to 24 weeks for patients with cirrhosis [6]. Additionally, all available DAA regimens require once- or twice-daily administration of multiple tablets provided by different manufacturers, which increases complexity and may present potential barriers to initiation of treatment and patient adherence. The single-tablet regimen of ledipasvir, NS5A inhibitor, combined with sofosbuvir (ledipasvir/sofosbuvir) for 8 or 12 weeks has proved to be highly effective and well tolerated in patients with genotype 1 HCV infection in phase 3 and real-world studies, with sustained virologic response rates of 94–99% [11–15].

Simple, once-daily, single-tablet regimens with short treatment durations and regimens without interferon or ribavirin and their associated side effects would benefit patients with chronic HCV infection in China. We conducted this phase 3b study to evaluate the safety and efficacy of ledipasvir/sofosbuvir for 12 weeks in Chinese patients with genotype 1 HCV infection, including those with cirrhosis and prior treatment experience.

## Materials and methods

### Design

This phase 3b, multicenter, open-label, single-arm study was conducted at 18 study centers in Mainland China between May 2016 and July 2017 (ClinicalTrials.gov NCT02021656). All patients received ledipasvir/sofosbuvir (90/400 mg) once daily by mouth for 12 weeks.

### Patients

Eligible patients were males and females over 20 years of age with confirmed genotype 1 chronic HCV infection and HCV RNA levels of at least 10^4^ IU/mL. Patients without cirrhosis or with Child–Pugh A compensated cirrhosis were eligible. Patients with hepatic decompensation, positive hepatitis B surface antigen, or human immunodeficiency virus were excluded. Detailed inclusion and exclusion criteria are provided in Supplement 1.

### Assessments

Screening, on-treatment, and posttreatment study assessments are outlined in Supplemental Table 1. Unless otherwise specified, genotyping and HCV RNA and clinical laboratory assessments were conducted at Covance Central Laboratory Services (Shanghai, China). Cirrhosis was confirmed by either liver biopsy (Metavir score = 4 or Ishak score ≥ 5) or FibroScan^®^ with a result > 12.5 kPa.

**Table 1:** Baseline demographics and disease characteristics

Characteristic	Treatment naive (*n* = 106)	Treatment experienced (*n* = 100)	Overall (*n* = 206)
Mean age (range), years	46 (23−70)	49 (21−72)	47 (21−72)
Age ≥ 65 years, *n* (%)	8 (8)	9 (9)	17 (8)
Male, *n* (%)	56 (53)	47 (47)	103 (50)
Mean BMI (range), kg/m^2^	23.3 (14−32)	23.6 (16−34)	23.4 (14−34)
HCV genotype 1b, *n* (%)	106 (100)	100 (100)	206 (100)
Compensated cirrhosis, *n* (%)	17 (16)	15 (15)	32 (16)
IL28B genotype, *n* (%)			
CC	84 (79)	73 (73)	157 (76)
CT	22 (21)	25 (25)	47 (23)
TT	0	2 (2)	2 (1)
HCV RNA			
Mean (SD), log_10_ IU/mL	6.2 (1)	6.4 (1)	6.3 (1)
≥ 800,000 IU/mL, *n* (%)	83 (78)	87 (87)	170 (83)
ALT > 1.5 × ULN, *n* (%)	48 (45)	37 (37%)	85 (41%)
Response to prior HCV treatment			
Relapse/breakthrough	NA	47 (47)	47 (23)
Non-response	NA	25 (25)	25 (12)
IFN intolerant	NA	28 (28)	28 (14)

*BMI* body mass index, *ALT* alanine aminotransferase, *eGFR* estimated glomerular filtration rate, *IFN* interferon, *ULN* upper limit of the normal range

Blood samples to determine serum levels of HCV RNA were collected prior to initiation of ledipasvir/sofosbuvir, every 2 weeks during treatment, and at posttreatment Weeks 4, 12, and 24. The COBAS^®^ AmpliPrep/COBAS^®^ TaqMan^®^ HCV Quantitative Test, v2.0 (Roche Diagnostics, Basel, Switzerland) was used to quantify HCV RNA; the lower limit of quantitation of the assay is 15 IU/mL. To evaluate drug-class RASs, the HCV NS5A and NS5B coding regions were deep sequenced for all patients at baseline and for any patients with virologic failure at the time of failure (Wuxi Genome Center, Shanghai, China) and evaluated using the Basic Local Alignment Search Tool (BLAST). All RASs that exceeded a 15% cut-off were reported. Safety assessments included monitoring of adverse events and clinical laboratory tests at all on-treatment visits and the posttreatment Week 4 visit, physical examinations at baseline and Week 12, vital sign measurements at all on-treatment and posttreatment visits, and ECGs at baseline, Week 1, and Week 12. Adverse events were coded using the Medical Dictionary for Regulatory Activities (MedDRA), version 20.0.

### Statistical analyses

The primary efficacy endpoint was SVR12 (i.e., HCV RNA less than the lower limit of quantitation 12 weeks after completing treatment). Point estimates and 2-sided 95% exact confidence intervals were constructed by the Clopper–Pearson method using the binomial distribution [16] by prior HCV treatment status (i.e., treatment naive or treatment experienced) and overall for the full analysis set (all enrolled patients who received study drug). The SVR12 rate for treatment-naive patients was compared with a historical SVR rate of 57% (i.e., treatment-naive Chinese patients with genotype 1 HCV who had been treated with pegylated interferon plus ribavirin for 48 weeks) [17, 18], using a 2-sided exact 1-sample binomial test. Safety data were analyzed for the safety analysis set (all patients who received study drug). Adverse events and laboratory abnormalities were summarized by patient count and proportion. All statistical summaries and analyses were conducted using SAS^®^ Software, Version 9.4 (SAS Institute Inc., Cary, NC, USA).

### Study oversight

This study was approved by an independent ethics committee at each participating site and was conducted in compliance with the principles of the Declaration of Helsinki, Good Clinical Practice guidelines, and local regulatory requirements. The study was designed and conducted according to the protocol by the sponsor (Gilead Sciences) in collaboration with the academic investigators. The sponsor collected the data, monitored the study conduct, and performed the statistical analyses. An independent data and safety monitoring committee reviewed the progress of the study. The investigators, participating institutions, and sponsor agreed to maintain confidentiality of the data. All authors had access to the data and assumed responsibility for the integrity and completeness of the data and analyses reported.

## Results

### Demographics and baseline characteristics

Of 223 screened patients, 206 patients were enrolled and received at least 1 dose of ledipasvir/sofosbuvir, including 106 (51%) treatment-naive and 100 (49%) treatment-experienced patients (Table 1); demographics and baseline disease characteristics were similar for treatment-naive and treatment-experienced patients. The mean age of patients was 47 years, 50% were male, and 16% had cirrhosis. All of the patients had confirmed genotype 1b HCV infection, and the majority (83%) had baseline HCV RNA ≥ 800,000 IU/mL and the CC IL28B allele (76%).

### Efficacy

All of the 206 patients achieved SVR12 (100%; 95% CI 98–100%) following 12 weeks of once-daily treatment with ledipasvir/sofosbuvir (Table 2). All treatment-naive patients achieved SVR12 (100%; 95% CI 97–100%), which was superior to the historical sustained virologic response rate of 57% following 48 weeks of pegylated interferon plus ribavirin (*p* < 0.001). Plasma levels of HCV RNA declined rapidly with treatment, with 99% of patients having HCV RNA less than the lower limit of quantitation after 4 weeks of treatment, and 100% of patients having HCV RNA less than the lower limit of quantification after 8 weeks of treatment. As all patients achieved SVR12, there was no impact of host or viral factors that have historically been predictive of or associated with lower sustained virologic response rates (e.g., older age, prior HCV treatment, high body mass index, cirrhosis, high viral load, non-CC IL28B allele).

**Table 2:** Virologic response rates during and after treatment with LDV/SOF

Response	Treatment naive (*n* = 106)	Treatment experienced (*n* = 100)	Overall (*n* = 206)
HCV RNA < LLOQ			
During treatment, *n*/*N* (%)			
95% CI			
At week 2	82/106 (77)	74/100 (74)	156/206 (76)
	68–85	64–82	69–81
At week 4	105/106 (99)	99/100 (99)	204/206 (99)
	95–100	95–100	97–100
At week 12	106/106 (100)	100/100 (100)	206/206 (100)
	97–100	96–100	98–100
Posttreatment, *n*/*N* (%)			
95 CI			
At week 4	106/106 (100)	100/100 (100)	206/206 (100)
	97–100	96–100	98–100
At week 12	106/106 (100)	100/100 (100)	206/206 (100)
	97–100	96–100	98–100
*p* value (SVR12 vs 57%)	< 0.001	–	–

The exact 95 CI for the proportion was based on the Clopper–Pearson method. The *p* value for the comparison of the SVR12 rate versus 57% was based on a 2-sided, 1-sample, binomial exact test

*CI* confidence interval, *LLOQ* lower limit of quantitation

### Resistance-associated substitutions

Twenty-nine (14%) of 206 patients had baseline NS5A RASs: 19 of 106 treatment-naive patients and 10 of 100 treatment-experienced patients. All of these 29 patients had a single Y93H RAS. Three of 205 evaluable patients (1%) had baseline NS5B RASs: 1 treatment-naive patient with N142T + L159F, and 2 treatment-experienced patients with L159F. All patients with pretreatment NS5A or NS5B RASs achieved SVR12.

### Safety

Overall, ledipasvir/sofosbuvir was well tolerated, and no patients prematurely discontinued study treatment (Table 3). Three patients (1%) had a serious adverse event (1 patient each with epicondylitis, asthma, and bone contusion). All serious adverse events were assessed as unrelated to study treatment. All adverse events were mild or moderate in severity, and no severe or life-threatening adverse events were reported. The most common adverse events were viral upper respiratory tract infection (17%), upper respiratory tract infection (14%), and cough (6%).

**Table 3:** Adverse events and grade 3 or 4 laboratory abnormalities frequency and severity

	LDV/SOF 12 weeks overall (*n* = 206)
Adverse events^a^	
Any adverse event, *n* (%)	120 (58)
Adverse event reported for ≥ 5% of patients	
Viral upper respiratory tract infection	36 (17)
Upper respiratory tract infection	28 (14)
Cough	12 (6)
Treatment-related adverse event	25 (12)
Grade 3 (severe) or 4 (life-threatening) adverse event	0
Serious adverse event^b^	3 (1)
Adverse event leading to premature discontinuation of study drug	0
Adverse event leading to interruption of study drug	0
Death	0
Grade 3 and 4 laboratory abnormalities^a^	5 (2)
Any Grade 3 laboratory abnormality, *n* (%)	5 (2)
Platelets (decrease)	1 (0.5)
Alanine aminotransferase (increase)	2 (1)
Serum glucose (hyperglycemia)	2 (1)
Any Grade 4 laboratory abnormality, *n* (%)	0

^a^Includes adverse events that occurred from the date of the first dose of study drug through 30 days after the date of the last dose of study drug (i.e., treatment emergent)

^b^Includes 1 event each of epicondylitis, asthma, and bone contusion

Five patients (2%) had grade 3 laboratory abnormalities (Table 3). The only grade 3 laboratory abnormalities observed in two patients were hyperglycemia and increased alanine aminotransferase. Both of the patients with hyperglycemia had a history of diabetes and elevated glucose at baseline, and both of the patients with Grade 3 increased alanine aminotransferase had a history of fatty liver and elevated alanine aminotransferase at baseline.

## Discussion

In this study, 100% of the Chinese patients with genotype 1 HCV infection achieved SVR following 12 weeks of once-daily treatment with ledipasvir/sofosbuvir, a short-duration, interferon- and ribavirin-free single-tablet regimen. Response rates did not differ for patients with prior treatment experience, compensated cirrhosis, pretreatment RASs, or those older than 65 years. The high SVR rates achieved by Chinese patients, as well as the lack of an impact of host or viral factors on response rates, are comparable to results observed in clinical trials conducted in other Asian countries and regions (SVR12 98–100%) and in the United States (US) and the European Union (EU) (SVR12 94–99%) overall and across a broad range of patient subgroups, including patients with prior HCV treatment experience and those with compensated cirrhosis [11–15, 19–21].

The presence of NS5A or NS5B RASs did not impact sustained virologic response. All of the patients with baseline NS5A RASs (14%) had a single Y93H RAS, and all achieved SVR12. While Y93 variants are known to confer resistance to NS5A inhibitors, ledipasvir/sofosbuvir is less affected by Y93 variants in vivo compared with other NS5A inhibitors [22]. Notably, the presence of NS5A RASs is associated with lower sustained virologic response rates in patients with genotype 1a compared with genotype 1b HCV infection [23], and all of the Chinese patients in this study had genotype 1b HCV. The 3 patients with NS5B RASs achieved SVR12. These results suggest that resistance testing prior to treatment with ledipasvir/sofosbuvir will have no clinical benefit in China, which is consistent with the current approved usage of ledipasvir/sofosbuvir in the US and in the EU [24, 25].

Ledipasvir/sofosbuvir was well tolerated by Chinese patients; adverse events were similar in type and rate as those reported for patients treated with 12 weeks of ledipasvir/sofosbuvir in other Asian countries and regions [19–21], with a numerically lower incidence compared with patients in the US and EU [11–15]. All adverse events were mild or moderate in severity; there were few serious adverse events, and no interruption or discontinuation of ledipasvir/sofosbuvir due to adverse events. Relatively high rates of viral upper respiratory tract infections (17%), upper respiratory tract infections (14%), and cough (6%) were reported. In China, acute respiratory tract infections, attributed primarily to influenza viruses, typically increase during the winter months [26, 27]. The majority of upper respiratory tract infections in this study occurred from December 2016 through March 2017, and the high rates may reflect the time of year that the study was conducted.

The incidence of Grade 3 laboratory abnormalities was low, and no clinically meaningful laboratory abnormalities were observed.

The efficacy, resistance, and safety results for HCV-infected patients in China who were treated with ledipasvir/sofosbuvir in this study can be considered confirmatory of the international experience with ledipasvir/sofosbuvir. Furthermore, the results from international clinical trials and real-world experience can be extrapolated to the broader HCV-infected population in China.

This study was limited where only the patients with genotype 1b HCV were enrolled, although genotype 1a patients were also eligible. Another limitation is the lack of a control group; however, the adverse event profile in this study is similar to what has been observed in other studies and is reflective of the ledipasvir/sofosbuvir safety profile [11–15].

In conclusion, treatment with ledipasvir/sofosbuvir single-tablet regimen for 12 weeks was well tolerated and resulted in 100% SVR12 in treatment-naive and treatment-experienced Chinese patients with genotype 1 HCV infection without cirrhosis or with compensated cirrhosis.

## Electronic supplementary material

Below is the link to the electronic supplementary material.

Supplementary material 1 (DOCX 32 kb)
