# Supplementary material for: Ledipasvir/sofosbuvir for treatment-naive and treatment-experienced Chinese patients with genotype 1 HCV: an open-label, phase 3b study
Source: Hepatol Int. 2018 Apr 10;12(2):126–32. doi: 10.1007/s12072-018-9856-z (PMC5904238; doi:10.1007/s12072-018-9856-z)
Supplement: Supplementary file 1 — Supplementary material 1 (DOCX 32 kb) [file 12072_2018_9856_MOESM1_ESM.docx]

Supplemental Appendix to:

**Ledipasvir/sofosbuvir for treatment-naive and treatment-experienced Chinese patients with genotype 1 HCV: an open-label, phase 3b study**

Lai Wei, Qing Xie, Jin Lin Hou et al.

**Supplement 1: Detailed inclusion/exclusion criteria**

The study population in Mainland China consisted of subjects who met all of the inclusion criteria and none of the exclusion criteria.

Inclusion criteria

1. Willing and able to provide written informed consent
2. Male or female, age ≥ 20 years
3. Body weight ≥ 40 kg
4. HCV RNA ≥ 10^4^ IU/mL at screening
5. Could be categorized as HCV treatment naive or treatment experienced as defined below:
   1. HCV treatment naive was defined as no prior exposure to any interferon, or other approved or experimental HCV-specific direct-acting antiviral
   2. HCV treatment experienced with medical records that included sufficient detail of prior interferon-based treatment to allow for categorization of prior response as either:
      1. Intolerant: Subject discontinued treatment due to development or significant worsening of an adverse event associated with HCV treatment.
      2. Nonresponse: Subject did not achieve undetectable HCV RNA levels while on treatment.
      3. Relapse/Breakthrough: Subject achieved undetectable HCV RNA levels during treatment or within 4 weeks of the end of treatment, but did not achieve a sustained virologic response.
6. Treatment-experienced subjects must not have discontinued prior treatment due to noncompliance with treatment.
7. Genotype 1 HCV at screening as determined by the central laboratory. Any nondefinitive results excluded the subject from study participation.
8. Confirmation of chronic HCV infection documented by either:
   1. A positive anti-HCV antibody test or positive HCV RNA test or positive HCV genotyping test at least 6 months prior to the baseline/Day 1 visit

OR

- 1. A liver biopsy performed prior to the baseline/Day 1 visit with evidence of chronic HCV infection

1. Cirrhosis determination (up to 20% of subjects enrolled in the study may have had compensated cirrhosis)
   1. Cirrhosis was defined as meeting any 1 of the following criteria:
      1. Liver biopsy showing cirrhosis (eg, Metavir score = 4 or Ishak score ≥ 5)
      2. Fibroscan indicative of cirrhosis as evidenced by a result > 12.5 kPa
   2. Absence of cirrhosis was defined as any 1 of the following:
      1. Liver biopsy within 2 years prior to screening showing absence of cirrhosis
      2. Fibroscan within 6 months prior to baseline/Day 1 with a result of ≤ 12.5 kPa
2. Liver imaging within 6 months of baseline/Day 1 to exclude hepatocellular carcinoma was required for subjects with cirrhosis
3. Screening electrocardiogram without clinically significant abnormalities
4. Subjects must have the following laboratory parameters at screening:
   1. Alanine aminotransferase ≤ 10 × the upper limit of normal
   2. Aspartate aminotransferase ≤ 10 × the upper limit of normal
   3. Direct bilirubin ≤ 1.5 × the upper limit of normal
   4. Platelets ≥ 50,000/µL
   5. Hemoglobin A1c ≤ 8.5%
   6. Creatinine clearance ≥ 50 mL/min calculated by the Cockcroft‑Gault equation
   7. Hemoglobin ≥ 11 g/dL for female subjects and ≥ 12 g/dL for male subjects.
   8. Albumin ≥ 3g/dL
   9. International normalized ratio ≤ 1.5 × the upper limit of normal, unless subject had known hemophilia or was stable on an anticoagulant regimen affecting nternational normalized ratio
5. Females of childbearing potential as defined in the study protocol must have had a negative serum pregnancy test at screening and a negative urine pregnancy test at baseline/Day 1 prior to enrollment.
6. Male subjects and female subjects of childbearing potential who engaged in heterosexual intercourse agreed to use protocol-specified methods of contraception, as described in Appendix 4 of the study protocol.
7. Subject was in generally good health, with the exception of chronic HCV infection, as determined by the investigator.
8. Subject was able to comply with the dosing instructions for study drug administration and able to complete the study schedule of assessments.

Exclusion criteria

1. Current or prior history of any of the following:
   1. Clinically significant illness (other than HCV) or any other major medical disorder that may have interfered with subject treatment or assessment or compliance with the protocol; subjects who were under evaluation for a potentially clinically significant illness (other than HCV) were also excluded.
   2. Gastrointestinal disorder or postoperative condition that may have interfered with the absorption of the study drug
   3. Difficulty with blood collection and/or poor venous access for the purposes of phlebotomy
   4. Clinical hepatic decompensation (ie, ascites, encephalopathy, or variceal hemorrhage)
   5. Solid organ transplantation
   6. Significant pulmonary disease, significant cardiac disease, or porphyria
   7. Psychiatric hospitalization, suicide attempt, and/or a period of disability as a result of psychiatric illness within the last 5 years. Subjects with psychiatric illness (without the prior mentioned conditions) that was well controlled on a stable treatment regimen for at least 12 months prior to baseline/Day 1 or had not required medication in the last 12 months may have been enrolled.
   8. Malignancy within the 5 years prior to screening, with the exception of specific cancers that are cured by surgical resection (eg, basal cell skin cancer); subjects under evaluation for possible malignancy were not eligible.
   9. Significant drug allergy, such as anaphylaxis or hepatotoxicity
2. If treatment naive, prior exposure to approved or experimental HCV direct-acting antivirals; if treatment-experienced, prior exposure to approved or experimental HCV-specific direct-acting antivirals other than NS3/4A protease inhibitors
3. Pregnant or nursing females
4. Chronic liver disease of a non‑HCV etiology (eg, hemochromatosis, Wilson’s disease, alpha‑1 antitrypsin deficiency, cholangitis)
5. Infection with hepatitis B virus or human immunodeficiency virus
6. Donation or loss of more than 400 mL blood within 2 months prior to baseline/Day 1
7. Use of any prohibited concomitant medications as described in
8. Administration of interferon or any HCV-directed treatment, other than the study drug, was prohibited from 12 weeks prior to screening until completion of the final posttreatment follow‑up visit
9. Chronic use of systemically administered immunosuppressive agents (eg, prednisone equivalent > 10 mg/day); azathioprine; or monoclonal antibodies, such as infliximab
10. Known hypersensitivity to ledipasvir, sofosbuvir or formulation excipients

**Supplemental Table 1: Screening, On-Treatment, and Posttreatment Follow-Up Assessments**

| **Clinical Assessment** | **Screening^a^ (Day -28 to Day -1)** | **Baseline/ Day 1^b^** | **Study Week (± 3 Days)** | | | | | | | | **ET** |
| --- | --- | --- | --- | --- | --- | --- | --- | --- | --- | --- | --- |
|  |  |  | **1** | **2** | **4** | **6** | **8** | **10** | **12** | |  |
| Informed Consent | X |  |  |  |  |  |  |  |  |  | |
| Determine Eligibility | X | X |  |  |  |  |  |  |  |  | |
| Medical History | X |  |  |  |  |  |  |  |  |  | |
| Physical Examination | X | X |  |  |  |  |  |  | X | X | |
| Liver Imaging (Only Subjects with Cirrhosis) | X |  |  |  |  |  |  |  |  |  | |
| Height | X |  |  |  |  |  |  |  |  |  | |
| Weight | X | X |  |  |  |  |  |  | X | X | |
| Vital Signs^c^ | X | X | X | X | X | X | X | X | X | X | |
| 12-Lead ECG^d^ | X | X | X |  |  |  |  |  | X | X | |
| AEs | X | X | X | X | X | X | X | X | X | X | |
| Concomitant Medications | X | X | X | X | X | X | X | X | X | X | |
| Pregnancy Prevention Counseling |  | X |  |  |  |  |  |  | X | X | |
| Health-Related QoL |  | X |  | X | X |  | X |  | X | X | |
| Hematology and Chemistry | X | X | X | X | X | X | X | X | X | X | |
| Coagulation | X | X |  |  |  |  |  |  | X | X | |
| Serum HCV RNA | X | X | X | X | X | X | X | X | X | X | |
| Single PK Sample |  |  | X | X | X | X | X | X | X | X | |
| Viral RNA Sequencing/ Phenotyping Sample (Plasma)^e^ |  | X | X | X | X | X | X | X | X | X | |
| Archive Sample ^h^ |  | X |  |  |  |  |  |  | X | X | |
| Serum or Urine Pregnancy Test^i^ | X | X |  |  | X |  | X |  | X | X | |
| Urinalysis | X |  |  |  |  |  |  |  |  |  | |
| IL28B Genotype, HCV Genotype | X |  |  |  |  |  |  |  |  |  | |
| HCV Ab, HIV Ab, and HBsAg | X |  |  |  |  |  |  |  |  |  | |
| HbA_1c_, TSH | X |  |  |  |  |  |  |  |  |  | |
| Review of Study Drug Adherence and Drug Accountability^f^ |  |  | X | X | X | X | X | X | X | X | |
| Study Drug Dispensing^g^ |  | X |  |  | X |  | X |  |  |  | |

Ab = antibody; AE = adverse event; ECG = electrocardiogram; ET = early termination; HbA_1c_ = hemoglobin A_1c_; HBsAg = hepatitis B virus S antigen; HIV = human immunodeficiency virus; PK = pharmacokinetics; QoL = quality of life; TSH = thyroid-stimulating hormone

1. The screening window may have been extended to 42 days for subjects requiring liver biopsy or additional HCV genotype testing.
2. Baseline/Day 1 assessments were performed prior to dosing.
3. Vital signs included resting blood pressure, pulse, respiratory rate, and body temperature.
4. Subjects were required to rest in a supine position for ≥ 5 minutes prior to ECG recording. The investigator (or qualified designee) reviewed the ECG traces for gross abnormalities in real time.
5. Plasma samples were collected and stored for potential HCV sequencing and other virology studies.
6. Study medication and dosing diary were reconciled at every postbaseline visit by the investigator or designee to monitor the subject’s adherence to the medication regimen.

g Study drug was dispensed according to interactive web response system directions. Subjects were instructed to return all bottles of study drug in the original container at every postbaseline study visits through the end of treatment.

h Archive plasma samples were collected at the baseline/Day 1 and end of treatment visits for subjects who did not opt out of sample collection.

i Only female subjects of childbearing potential were assessed for pregnancy. In the event of a positive urine test, a serum pregnancy test was performed.
